# The Effect of Dietary Halloysite Supplementation on the Performance of Broiler Chickens and Broiler House Environmental Parameters

**DOI:** 10.3390/ani11072040

**Published:** 2021-07-08

**Authors:** Małgorzata Nadziakiewicz, Marcin Wojciech Lis, Piotr Micek

**Affiliations:** 1 CIECH Sarzyna S.A., ul. Chemików 1, 37-310 Nowa Sarzyna, Poland; malgorzatanadziakiewicz@gmail.com; 2 Department of Zoology and Animal Welfare, Faculty of Animal Science, University of Agriculture in Krakow, Mickiewicza 24/28, 30-059 Krakow, Poland; marcin.lis@urk.edu.pl; 3 Department of Animal Nutrition and Biotechnology and Fisheries, Faculty of Animal Science, University of Agriculture in Krakow, al. Mickiewicza 24/28, 30-059 Krakow, Poland

**Keywords:** broiler chicken, halloysite, additive, performance, environmental quality

## Abstract

**Simple Summary:**

Dietary supplementation of clays and clay minerals can moderate the processes of digestion and nutrient absorption, promoting animal growth and vitality. The mode of action of this process mostly relies on epithelium protection, preventing both irritation and lesions by binding pathogenic bacteria and toxins. Clays also help to reduce the volume of harmful gastrointestinal gases and other pollutants present in broiler facilities. However, minerals differ in structure and physico-chemical properties, which influences the results of their use in animal production. Therefore, the aim of this study was to determine the effect of dietary supplementation of halloysite—one of the aluminosilicates included in the group of clay minerals (kaolinite group)—on the mortality, production indices, and water consumption of broiler chickens, as well as on chosen broiler house environmental parameters. The addition of halloysite to the diet of broiler chickens in the amount of 1% resulted in a favourable reduction in feed consumption per unit of body weight gain and higher utilisation of crude protein, which in turn led to improved fattening results. Moreover, halloysite addition reduced the consumption and excretion of water by chickens and the ammonia content in the air, thus improving the living conditions in the broiler house.

**Abstract:**

The aim of the study was to determine the effect of supplementing broiler chickens’ diets with halloysite on daily body weight gain (BWG), feed conversion ratio (FCR), daily water consumption (DWC), and some broiler house hygiene parameters. The trial was conducted on 18,000 broiler chickens divided into two groups throughout the 42-day (D) rearing period. The birds were fed complete diets without (group C) or with halloysite addition (1%, group E) from D8 of rearing. No difference in the mortality rate was observed between groups C and E. Birds from group E had a tendency (0.05 < *p* < 0.10) towards a higher body weight at D32 and D42, a higher BWG, and a lower FCR compared to group C during the entire rearing period. Average DWC differed only in the finisher period, with a tendency towards lower overall DWC in group E. The concentration of ammonia in the air from D21 to D35 was increased more than 5-fold in group C but only 1.5-fold in group E. In conclusion, the use of halloysite as a feed additive in the diet of broiler chickens resulted in a reduction in feed consumption per unit of BWG and higher utilisation of crude protein, which led to improved environmental conditions.

## 1. Introduction

Chicken meat consumption is increasing significantly. For this reason, during the last a few decades, many efforts have been made to increase efficiency in broiler production. In this context, one promising way to decrease production costs and increase feed’s nutritional effectiveness is the use of feed additives such as aluminosilicate minerals [1]. These materials are not digested, but bind toxic metabolites and mycotoxins, moving them through the digestive tract, without detriment to the animal’s body [2,3]. It has been shown that clays and the clay minerals included in the aluminosilicate group may increase animal growth and vitality, strengthen immune functions, and prevent different kinds of diseases, as well as improving meat and derived final product quality [4,5].

Dietary supplementation of clays can moderate the processes of digestion and nutrient absorption, promoting a longer retention time of the digesta, allowing its greater digestion and absorption to improve the performance of farm animals [1,6]. The mode of action of this process mostly relies on epithelium protection by forming a colloid that covers the intestinal mucosa, preventing both irritation and lesions, and by binding the bacteria and toxins to eliminate them along with faeces [3,6]. Including clays in feed may also cause an increase in villus height and in the villus height to crypt depth ratio, which in turn increases the surface area of the gastrointestinal tract, thus increasing nutrient digestibility [7].

The excess moisture of excreta is the main reason for the presence of numerous pathogen-transmitting microorganisms in birds’ breeding environments. One way to control litter moisture is the application of water-adsorbing substances to the feed, which forms a complex with water. Actually, in the gastrointestinal tract, clays have the capacity to absorb water in an amount many times greater than their weight, preventing it from remaining free in the excreta. It has been demonstrated [8,9,10] that by reducing the rate of digesta passage and colloid formation, clay positively improves the consistency of chicken faeces. It has also been shown that clays help to reduce the volume of harmful gastrointestinal gases, off-site transport of odours, and other broiler facility pollutants [4,11].

On the other hand, Ullman et al. [11] emphasised that some studies have not found clay additives to have a significant effect on excreta moisture. Moreover, clays may bind not only noxious substances but also some nutrients such as vitamins, growth promoter additives, or trace elements, causing a nutritional imbalance for animals by forming non-absorbable complexes [4,7]. Therefore, the average incorporation of clays into broiler chicken diets reported in the scientific literature usually varies between 0.5 and 3%, but the safe levels at which clays can be added to the diet to promote only their beneficial effects have not yet been well elucidated [12].

Minerals differ in structure and physical and chemical properties, which influences the results of their use in animal production [13]. In this context, the beneficial effect of a clay’s use as a feed additive may depend not only on its origin or level of incorporation in the diet, but also on other properties related to the deposit from whence it came and any pollutants found there.

Various clays have been used in research trials as feed additives, including kaolinite minerals [14,15,16,17]. Individual kaolin deposits vary, particularly in their physical properties, which in turn influences kaolin end uses. Therefore, the results of studies carried out on the kaolin from a certain deposit cannot be extrapolated to any other kaolin deposit [18]. Generally, kaolin group minerals are 1:1 layer phyllosilicates. One of them, halloysite, consists of nanotubes (HNTs) which form in multiple rolled layers composed of a sheet of corner-sharing SiO_4_ tetrahedrals bonded to edge-sharing AlO_6_ octahedrals. This structure of halloysite has negative charges on its external surface, positive charges on its inner lumen surface, and both negative and positive charges at its edges. The hydroxyl groups at the surfaces and edges of HNTs provide a useful opportunity for modification with various organic compounds [19]. The world’s largest deposit of this mineral is located in Dunino (Poland) and characterised by a specific platy-tubular structure with a ratio of halloysite HNTs (7 Å) to kaolinite of 66:34 and a specific surface area of 65–85 m^2^/g.

The aim of this study was to determine the effect of supplementing broiler chickens’ complete diets with halloysite on mortality, average daily body weight gain (BWG), feed conversion ratio (FCR), and the daily water consumption (DWC) of broiler chickens, as well as on chosen broiler house environmental parameters.

## 2. Materials and Methods

### 2.1. Experimental Design

The trial was conducted during winter (January/February) on an experimental poultry farm in Zalesiany (Malopolska voivodeship, Poland, 49°52’14.1” N 20°12’40.4” E) in a broiler house with an area of 1000 m^2^, with a litter floor system. Prior to the experiment, the building and equipment were thoroughly cleaned and disinfected in accordance with the principles of veterinary biosecurity. Before the chicks were delivered, the hall was lined with bedding (wheat straw) and heated to an air temperature of 34 °C and a floor temperature of 28 °C.

Day-old Ross 308 broiler chicks (Aviagen EPI ltd, Poland) were randomly divided into two 9000-bird groups based on treatment: control (C) and experimental (E). Each group was separated into five pens (100 m^2^) with 1800 chicks per pen. The broiler chickens had constant access to water and feed throughout the rearing period, in which 4 types of nutritionally balanced complete diets (Table 1) were used, according to the age of the birds: starter (day of life (D) 1–7), grower I (D8–D19), grower II (D20–D32), and finisher (D33–D42). The complete feed granulated mixtures (adapting the composition and size of the granules to the rearing period) were prepared according to Cargill Feed & Nutrition standards (Minneapolis, MN, USA) and contained (in different proportions): wheat grain, maize grain, triticale grain, soybean meal, canola meal, sunflower meal, maize DDGS, dried swine red blood cells, fish meal, vegetable oil, raw soy lecithin, mineral–vitamin additives, endo-1,4-beta-ksylanase enzyme, phytase enzyme, preservatives (E330, E327, E238), salinomycin sodium, and an antioxidant (ethoxyquin). From D1 to D7, both groups of chickens were fed the same kind of feed, crumble starter, but from D8, group E began to receive the feed with 1% addition of halloysite (10 kg/ton of feed) from the Dunino deposit (Poland, 51°09′20″ N 16°05′12″ E). The halloysite mineral was naturally free of asbestos, dioxins, and heavy metals (according to the EU Register of Feed Additives, pursuant to Regulation (EC) No 1831/2003) and prepared in advance for use in animal nutrition (powder form). The additives were mixed in with the other ingredients of the complete feeds prior to granulation.

Feed and water were given ad libitum throughout the complete broiler production cycle, continuously tracking the amount of the feed (kg/day) and water consumption (l/day). The measurement of water consumption began on D8 (beginning of the halloysite additive’s use). The process of supplying the feed was conducted by means of a feeding system (via feed pipes), which was controlled automatically. The water quality met drinking water standards for humans (Polish standard, JL No. 61, 417). The heating and lighting programs were in accordance with Ross 308 broiler stock management [21].

### 2.2. Veterinary Treatments

The chicks were vaccinated against infectious bronchitis (IB) by the coarse drop method on D1 and D7 and against Gumboro disease (IBD) by a vaccine added to the water on D16 and D23. Moreover, on the 21st broiler rearing day, samples were taken from the litter surface to test for Salmonella spp., according to the formal sampling scheme [22]. Two pairs of boot swabs were used for sampling and the samples were taken by walking around in the poultry house such that all sections in the house were proportionally represented. One pair of boot swabs was used to cover around 50% of the area of the poultry house. On completion of sampling, the swabs were carefully removed from the boots, so as not to dislodge adherent material, and placed in a bag or pot and labelled. The samples were tested using the horizontal method for the detection, enumeration, and serotyping of *Salmonella* (according to ISO 6579-1:2017) at the laboratory of the Veterinary Hygiene Department of the Malopolska Veterinary Inspectorate in Krakow (Poland) (certificate No. 531 of the Polish Centre for Accreditation for the ISO/IEC 17025:2005 standard) and no *Salmonella* was found.

### 2.3. Measurements and Observations

#### 2.3.1. Production Indicators

The chicken body weight on the last day of each period (i.e., D7, 19, 32, and 42) was collected by sampling 100 randomly selected chickens (with an equal proportion of females and males) from each pen of each group. On these days, the measurement was taken at 8:00 a.m., before starting the feeders with the new type of feed. Throughout the whole period of rearing, the average daily water consumption (DWC) of each pen in each group was recorded. The feed conversion ratio (FCR) was calculated for each rearing period and the entire cycle according to the formula: FCR = feed intake (kg)/body weight gain (kg). The calculations were performed for each pen separately, taking into account the feed intake, the average weight of one chicken, and the number of chickens in the pen. Throughout the experiment, the flocks were inspected 3 times daily to supervise the welfare conditions and count cases of dead animals. Chicken mortality during rearing (ML) was calculated according to the formula: ML (%) = (number of dead individuals/number of set chicks) × 100.

#### 2.3.2. Environmental Measurements

Environmental measurements of air and litter, as well as sampling of manure and litter to determine their humidity and ammonia content, were carried out two times (on D28 and D35) at 15 similarly located points inside groups C and E (3 points per pen). Air temperature (T), relative humidity (RH), and velocity (V) were measured by multi-instrument type TA465 (TSI-Airflow Instruments Ltd., High Wycombe, UK), and cooling rate (CR) was measured with a certified Hills kata-thermometer (Kujawska Wytwórnia Termometrów, Poland; certificated by the Institute of Meteorology and Water Management—National Research Institute, Poland). Gas concentrations (carbon dioxide (CO_2_), ammonia (NH_3_), and hydrogen sulphide (H_2_S)) were measured with a Multi-Gas Detector Polytector II G750 (GfG Hauptsitz, Dortmund, Germany).

The temperature of the litter surface was measured with a DT822 pyrometer (Shenzhen Cheerman Technology Co., Ltd., Guangdong, China), randomly in 10 replications (1 per pen) on the surface of a circle with a radius of 1 m from the observer. The internal temperature of the litter was measured at a depth of approx. 3 cm, in parallel with two mercury thermometers placed at a distance of approx. 0.5 m from each other. The values were read approx. 15 min after the devices were immersed in the litter.

The samples of litter (three for each point) were collected in 100 mL airtight plastic containers and stored in the cooler (+4 °C) for the subsequent laboratory analyses. The humidity of the litter was determined by the weight method by comparing the weight of the fresh sample and the weight of the sample after drying at 105 °C for 48 h.

#### 2.3.3. Apparent Crude Protein Digestibility

The results regarding the chemical composition of feeds and excreta were used to calculate the coefficient of crude protein apparent digestibility (ACPD) using acid insoluble ash (AIA) as a digestibility indicator (the indicator method). The ACPD was calculated by the alpha-amino nitrogen (N-NH2) method [23], as modified by Barteczko et al. [24]. This method is based on the determination of alpha-amino groups in the faeces from undigested feed proteins. The first stage is the hydrolysis of feed protein and faeces in hydrochloric acid (HCl). Subsequently, the faeces samples are subjected to pressure distillation to remove the non-protein nitrogen fraction (mainly ammonia-NH_3_), which can positively react with ninhydrin and thus affect the results. Hydrolysates of the distilled faeces and the corresponding feed undergo a reaction during which free amino groups form a coloured complex with ninhydrin. Next, the excretion of faecal samples and corresponding feed samples were measured at the wavelength of 570 nm. The representative faeces samples were collected during the last 5 days of the grower (D28–D32) and finisher (D38–D42) periods of chicken rearing. Each day of sampling, fresh faeces from 10 locations of each pen was polled, resulting in five samples per group, which were then immediately submitted to the laboratory for chemical analysis.

### 2.4. Chemical Analysis

Prior to chemical analysis, air-dried samples of complete mixtures were ground to pass through a 0.75 mm sieve with a Pulverisette 15 Laboratory Cutting Mill (Fritsh GMBH, Idar-Oberstein, Germany) and analysed to determine the content of the dry matter (DM), crude ash, crude protein (CP), crude fat (CFat), and crude fibre (CF) using standard analytical procedures (procedure nos. 934.01, 942.05, 976.05, 920.39, and 962.09, for DM, ash, CP, CFat, and CF, respectively) [25]. Neutral detergent fibre was identified with heat-stable amylase (aNDF) [26]; acid detergent fibre (ADF; official method 973.18 [25]) and acid detergent lignin (ADL [27]) were identified using an Ankom^220^ Fiber Analyzer (Ankom Technology, Macedon, NY, USA). The starch content was determined via an enzymatic method [28]. The same procedures were used for chemical analyses of the manure and litter. AIA content in feeds and faeces was analysed gravimetrically as a proportion of a sample that is not hydrolysed by 72% sulphuric acid and is not subsequently volatilised upon the incineration of this residue [29].

The pH of the litter samples was determined using a pH-meter type N517 (Meratronik, Warsaw, Poland) in an aqueous solution created after 48 h of soaking the sample in distilled water. In the same aqueous solution, the concentration of ammonium cations (NH4+) was determined by the Conway colorimetric method [30].

### 2.5. Statistical Analysis

The normality of data distribution was tested using the Shapiro–Wilk test and variance homogeneity was established by the Levene test. To compare the means of groups C and E, an unpaired Student’s *t*-test was applied. The statistical model included the random effect of bird and the fixed effect of treatment (halloysite addition). Data were analysed as a randomised complete block design, in which the pen was considered a block. Data on environmental parameters were tested by the two-way ANOVA and the Tukey post hoc test. Data were analysed using SAS software (SAS Inst. Inc., Cary, NC, USA). Differences between means were considered significant at *p* < 0.05 and a tendency at 0.05 < *p* ≤ 0.10, unless otherwise stated. Data are presented as means and standard error of the mean (SEM).

## 3. Results

### 3.1. Production Indices

The content of DM and nutrients in the halloysite and compound feed is presented in Table 1. The starter diet did not contain halloysite, whereas feeds used in later stages of rearing differed slightly in their chemical composition due to the 1% addition of halloysite. Compared to group C, the feeds used in group E were characterised by a higher concentration of CA, which in turn decreased the concentration of other nutrients as an effect of dilution. Regardless of halloysite addition, as the birds aged, the diets contained less CP and NFC but more CFat and starch.

In the initial period of rearing (starter diet) in group E, a slightly higher mortality rate was observed compared to group C (0.49% vs. 0.38%; *p* = 0.417; Table 2). A similar relationship between nutritional groups was also found in later stages of rearing, but these differences were not statistically significant (*p* > 0.05). Similarly, no differences were found between the nutritional groups in the body weight of birds at days of life 1, 8, and 20. Tendencies towards higher body weight in birds from group E were observed at D33 (*p* =0.098) as well as at D42 (2690 vs. 2750; *p* = 0.056). The obtained difference (60 g) means an increase in the final body weight of birds from group E of 2.2%. A tendency towards increased differences between nutritional groups was observed during the finisher period, i.e., from D33 of fattening, in favour of group E.

Average DWC expressed as litres per day per group (Table 2) differed between nutritional groups only in the finisher period (D33–D42; *p* = 0.049). However, tendencies towards lower DWC (*p* = 0.077) in group E were observed for the overall period of chicken rearing. The apparent CP digestibility was higher in group E compared to group C by 7.5 percentage points (*p* = 0.001) in the grower II period (D20–D32). In the finishing period (D33–D42), the tendency (*p* = 0.055) towards higher CP digestibility in group E was noted.

**Figure 1:**
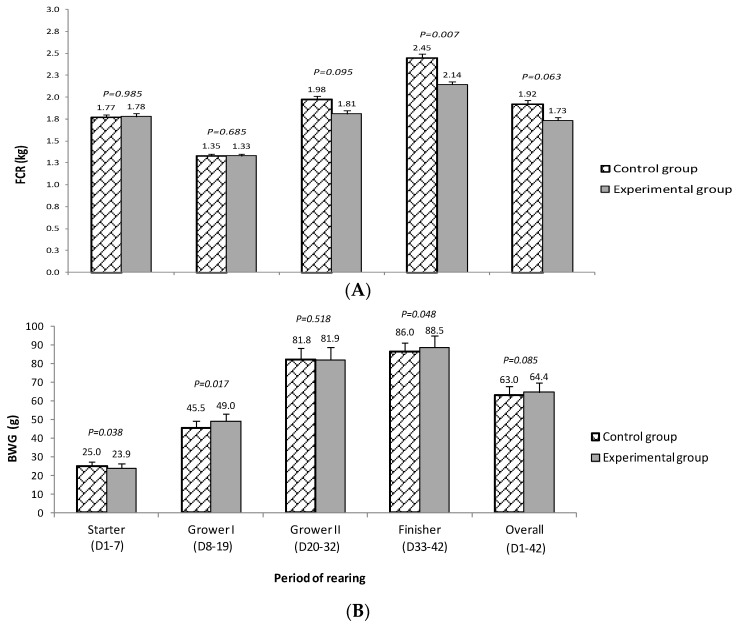
Feed conversion ratio (FCR (**A**)) and average daily body weight gain (BWG, g/d; (**B**)) of chickens fed complete mixtures without halloysite (control group) or with 1% of halloysite addition (experimental group).

Feed conversion ratio and average BWG is shown in Figure 1. The tendency towards lower FCR in birds from group E compared to group C was observed (*p* = 0.095) in the grower II period (D20−D32) as well as in the whole period of rearing (*p* = 0.063). The average FCR throughout the entire rearing period was 1.73 and 1.86, respectively (lower by 7.5% for group E). The differences in FCR between groups E and C increased gradually and reached the highest values in the finisher period (D33−D42; 2.14 vs. 2.45, respectively; *p* = 0.007). In the last 10 days, it was as much as 14.5% in favour of group E.

Except for the initial period (starter diet), animals from group E achieved numerically higher BWG compared to group C. The largest differences between the nutritional groups in favour of group E were observed in the grower I (D8−D19; 4.5 g/D; *p* = 0.017) and finisher periods (D33−D42; 2.5 g/D; *p* = 0.048). For the overall period of rearing, a tendency (*p* = 0.085) towards higher BWG among birds from group E was observed (64.4 g and 63 g for E and C groups, respectively).

### 3.2. Environmental Parameters in Broiler Houses

Both the air temperature (*p* = 0.147) as well as air velocity (*p* = 0.147) in groups E and C were similar during the experimental period (Table 3). The RH in group E was kept lower in comparison to C, respectively, 49 and 57% (*p* = 0.022) on D21; however, on D35, it significantly increased to 82% in both groups. In consequence, the cooling rate was lower in group E in comparison to group C on D21 (31 vs. 34.7 mWcm^−2^; *p* < 0.001) but not on D35 (41.9 vs. 38.8 mWcm^−2^; *p* = 0.139; Table 3). The concentrations of CO_2_ and NH_3_ in the air of the chicken house decreased during rearing (*p* < 0.001) and were 3079 vs. 3090 ppm CO_2_ (*p* = 0.968) and 7.2 vs. 4.4 ppm NH_3_ (*p* = 0.078) on D21 and 5250 vs. 5742 ppm CO_2_ (*p* = 0.116) and 10.9 vs. 23.2 ppm NH_3_ (*p* < 0.001) on D35 in groups E and C, respectively. However, comparing D21 to D35, the concentration of ammonia increased more than 5-fold in group C (*p* < 0.001) but only 1.5-fold (*p* > 0.05) in group E (Table 3). The H_2_S concentration in both of the assessed groups was below the sensitivity of the measurement method used.

The superficial litter temperature changed similarly to the air temperature, i.e., these parameters differed between groups (24.9 vs. 26.4 °C on average for groups E and C, respectively; *p* < 0.001) but not between D21 and D35 (*p* = 0.862). However, the internal litter temperature increased by around 2 °C at a depth of 3 cm and, moreover, was higher in group E than group C (28.6 vs. 27.9 on average; *p* = 0.085). The litter dry mass in both buildings ranged from 58.8 to 64.6% (*p* = 0.594); however, the contents of NH_3_ on D35 were 22.4 vs. 16.3 ppm (*p* = 0.064) in groups E and C, respectively (Table 3).

## 4. Discussion

The rapidly growing global population and the constant pursuit of greater efficiency in animal production have prompted copious research in which mineral additives have been scientifically tested. Clay minerals are at the centre of this interest due to their detoxifying/decontaminating properties. There are many studies using different clay group minerals as feed additives, which analyse their impact on poultry performance [13]. Prior to this study, however, there had still been few studies on the use of halloysite in broiler chickens’ nutrition.

According to Owen and Nodu [31], the addition of kaolin, from the clay group, to the broiler diet had no effect on the final body weight of birds. Similarly, Ouachem et al. [32] showed that the addition of kaolin at up to 30% of dry matter had no significant effect on the same parameter; however, the carcass yield increased with an increasing proportion of kaolin supplementation. These observations are consistent with our results, wherein chicken body weight was only slightly affected by halloysite addition, whereas carcass yield increased significantly, mainly due to the lower mass of the gastrointestinal tract [33].

The other parameters, such as FCR and BWG, have been widely used in the literature to reflect the effect of clay minerals on broiler chicken nutrition. The reduction of FCR, while achieving a higher BWG, indicates the real economic benefits of the additive. In this context, comparison of our results with the data from the literature may illustrate the relative benefits of halloysite over other minerals, although the breeding conditions and feed quality in these trials differed significantly. In our opinion, a better picture of the effects of halloysite addition to the diet was illustrated by comparing the percentage changes in the analysed parameters in relation to the control group than when comparing the numerical results. In the current research, the FCR calculated for the entire fattening period of 42 days was 7% lower for group E compared to group C. In turn, BWG was higher by 2.3%, which means lower feed consumption and higher final body weight of broilers.

Different experiments using various clay mineral additives in broiler diets have been described in the literature. Eser et al. [34], after a 42-day experiment, achieved 2.1 and 2.7% higher BWG using 0.5 or 1% sepiolite addition to the diets (respectively), compared to the control group. This level of BWG is similar to our results experimenting with halloysite. However, halloysite addition (1% as fed) caused a 7% reduction in FCR compared to the 2.2 and 2.7% (respectively) drops in FCR achieved in the compared studies. We hypothesise that halloysite’s beneficial effect could be due to the better utilisation of feed protein, as indicated by the 10.2% increase in the protein efficiency ratio (PER) index (data not shown). The positive effect of the mineral silicate’s supplementation on the ileal digestibility of energy and protein in broiler chickens has already been suggested by Safaei Katouli et al. [35]. In turn, Uzunoglu and Yalcin [17] showed a reduction in BWG of 1.7% and an increase in FCR of 2% when using sepiolite in the amount of 1.5% during 42 days of broiler rearing. The PER index decreased by 1.3% compared to the control group, which would indicate the inefficiency of increasing the share of sepiolite in the diet.

Wu et al. [14] tested the addition of 2% clinoptilolite to the diet of broiler chickens for 42 days. There were no differences in FCR, whereas BWG was 0.1% lower in the treatment group compared to the control. In another experiment of Wu et al. [36], the addition of 2% clinoptilolite to the diet lowered BWG by 5.2% and increased FCR by 4%. The addition of 2% clinoptilolite to the broiler diet was also tested in a study by Sacakli et al. [37]. This experiment was conducted in two combinations without or with phytase supplementation. In the first combination (without phytase), the treatment group was characterised by an increase in BWG of 2.4% and a decrease in FCR of 3.7%. In the treatment group with phytase, BWG decreased by 1.9%, with FCR higher by 1.3%. In turn, Karamanlis et al. [38], using 2% clinoptilolite, obtained an increase in BWG by as much as 6.9%, but FCR also increased by 5.9%.

The other clay mineral widely tested as a feed additive for broiler chicken is polygroskite. Li et al. [16] reported a 0.7% decrease in BWG and a 0.6% increase in FCR when a polygroskite additive was used at the level of 1%. Different combinations of polygorskite and yeast distributed simultaneously in this experiment indicated that the use of polygorskite as a feed additive in broiler nutrition was not economically justified. Similarly, bentonite, frequently used during industrial feed production as an anti-caking agent or a coagulant, is still the subject of research. Bouderoua et al. [39] have tested two kinds of bentonite—bentonite humidified in advance by water vapour and bentonite without previous humidification—both on two levels of incorporation into the diet, 2 or 5%, beginning on day 12 of the birds’ lives. Between days 14 and 42 in the treatment group with humidified bentonite on both levels of incorporation, an increase in BWG by 3.4 and 2.9%, respectively, was observed. The addition of this mineral did not bring any economic benefits due to the significant increase in FCR by 10.5 and 14.2%, respectively. In the experiment with 2% addition of bentonite without previous humidification, BWG increased by 4.9% and FCR by 4.9%. In the treatment group with 5% of bentonite without previous humidification, FCR decreased by 9.9%, but also BWG decreased as much as by 14.5%. In practice, this effect most likely leads to extending the fattening period to obtain the desired weight of birds, thus delaying the next fattening cycle, lowering the economic result of the farm. In no combination of experimental factors did both indicators, BWG and FCR, change in a favourable direction simultaneously. Therefore, such results disqualify the profitability of using bentonite as a feed additive for broilers. In our research, during the same period of fattening, halloysite supplementation caused an increase in BWG of 3.1% while FCR dropped by as much as 10.8%, which means higher final body weight achieved at reduced feed cost.

In the study of Cabuk et al. [40], the use of zeolite in the diet at the level of 1.5% or 2.5% for 42 days of broiler rearing was examined. In both treatment groups, BWG was lower by 7.8 and 4.9% and FCR was higher by 6.6 and 5.6%, respectively, compared to the control. A significant FCR reduction of 10 and 20% was achieved by Ani et al. [15] with 1 or 2% incorporation of a non-specified clay additive used in an experiment conducted from day 14 to 42 of bird fattening. BWG increased by 1.8% in the group with 1% of the additive, whereas 2% of the additive caused a decrease in BWG of 4.6%.

The aim of the research conducted by Safaei Katouli and Boldaji [12] was to determine the effect of kaolin, bentonite, or zeolite supplementation to the diet (1.5 or 3%) on broiler chicken performance. Among the three tested clay minerals, bentonite was the least effective as a feed additive, which was also demonstrated in the study of Bouderoua et al. [39]. The best result concerning performance indicators was achieved in the group fed with 3% kaolinite. Halloysite from the Dunino deposit used in the current study is from the kaolinite group of minerals. Therefore, some properties of halloysite and kaolinite are similar, especially regarding their nanotube structures, which determine the specific physico-chemical properties of a given mineral.

In our study, DWC depended on the period of rearing and treatment. Higher DWC in group C was observed between days of life 15 and 22 and from day 30 until the end of fattening. During these periods, the use of halloysite reduced water intake by the animals, which seems beneficial due to the reduction of litter moisture. This effect may have resulted from slowing down the flow of digesta in the birds’ gastrointestinal tracts. The reduced digesta flow rate probably caused a longer retention time of feed in the digestive tract (parameter not measured), and thus better digestion, while reducing the amount of excreted liquid faeces. Data on water intake by broiler chickens in digestibility trials with clay minerals are missing in the available literature; therefore, it is not widely discussed.

The thermal–humidity parameters in both chicken-houses were in line with the recommendations and provided for the thermal comfort of the birds [21]. However, on D21, the RH was 11 percentage points lower in the experimental group than in the control. This significant reduction in air saturation by water vapour in the facility might have resulted from the addition of halloysite into the feed. On D35, this effect was not visible, which may be related to the limited ventilation caused by low air temperatures outside (air temperature during this period at night fell below −20 °C). It also resulted in a significant increase in CO_2_ concentration, above the recommended 3500 ppm [21]. Simultaneously, if the ammonia concentration on D21 (after 13 days of the halloysite supplementation) was kept within recommended range in both groups [21], on D35, it was still truly low only in the chicken-house where the broilers were fed with the addition of halloysite. The reduction in the concentration of this gas can be related to the binding of ammonium ions in the substrate (an increase in N-NH_3_ in the litter was found). A similar effect was noted for *Yucca schidigera* and zeolite [40], as well as bentonite [41]. The reduction in ammonia emissions is a key issue as it affects both the health of handlers and the birds’ production results due to the specific anatomical structure of the respiratory system [42].

## 5. Conclusions

The use of halloysite as a feed additive in the diet of broiler chickens in an amount of 1% resulted in a favourable reduction in feed consumption per unit of body weight gain and a higher utilisation of crude protein, which in turn led to improved fattening results. Using halloysite as a feed additive reduced the consumption of water by chickens, which limited the amount of water excreted in faeces, thus significantly improving the living conditions in the broiler house by reducing the ammonia content in the air and in the litter. However, further research is needed to better define the mode of action of halloysite in the gastrointestinal tract of broiler chickens, including its different level of incorporation into the diet and differences found between deposits.

## Figures and Tables

**Table 1:** Chemical composition of compound feeds (% of dry matter).

Item	Starter	Grower I		Grower II		Finisher	
		C ^1^	E ^2^	C	E	C	E
Dry matter, %	91	89.7	90	88.1	88.4	90	89.7
Crude ash	6.4	5	6	4.9	6	4.3	5.2
Crude protein	24.3	23.6	23.4	22.8	22.2	21.6	21.3
Crude fat	4.9	4.5	3.9	6.7	6.2	9.6	9.1
Crude fibre	2.9	3.9	3.4	3.7	3.4	3.2	2.7
NDF	8.5	11.3	12	10.7	11.3	14.3	15.5
ADF	4.5	6.3	5.6	5.3	5.1	6.8	6.1
ADL	1.2	1.6	1.4	1.5	0.7	2.1	1.4
Starch	45.3	47.4	45.9	50	48.7	51.3	49.2
NFC ^3^	55.9	55.5	54.7	55	54.4	50.9	48.4
Calculated composition:							
ME, MJ/kg ^4^	12.8	13.1	13	13.4	13.3	13.6	13.4
Calcium	1.01	0.71	0.72	0.62	0.63	0.50	0.51
Phosphorus	0.69	0.61	0.61	0.53	0.53	0.47	0.47
Sodium	0.18	0.17	0.17	0.16	0.17	0.16	0.16
Lysine	1.48	1.37	1.37	1.30	1.30	1.29	1.29
Methionine	0.64	0.60	0.60	0.60	0.60	0.60	0.60

^1^ Control group fed complete mixtures without halloysite; ^2^ Experimental group fed complete mixtures with 1% halloysite added; ^3^ Non-fibre carbohydrates; NFC = 100 − (NDF + crude protein + crude fat + crude ash); ^4^ Metabolisable energy calculated according to the European Table [20] as a sum of the ME content of components.

**Table 2:** Mortality and body weight of broiler chicken, water consumption per group, and apparent crude protein (CP) digestibility of grower II (20–32) and finisher (33–42 days of life) complete feed.

Item	Treatment ^1^		*p*-Value	SEM
	C	E		
Mortality, number of animals (%)				
D1–7	69 (0.38)	88 (0.49)	0.417	1.6
D8–19	166 (0.92)	177 (0.98)	0.558	0.8
D20–32	81 (0.55)	95 (0.59)	0.398	0.6
D33–42	69 (0.47)	66 (0.41)	0.847	0.8
Overall	385 (2.32)	426 (2.47)	0.186	0.6
Body weight (n = 500 per group), g				
D1	45	45	0.989	0.1
D8	220	212	0.822	12
D20	766	800	0.155	36.1
D33	1830	1865	0.098	63.5
D42	2690	2750	0.056	89.4
Average daily water consumption (n = 5 per group), L/d/group				
D1–7	327	338	0.826	24.3
D8–19	1039	1014	0.870	72.9
D20–32	2015	2031	0.882	52.5
D33–42	2561	2443	0.049	33.7
Overall	66,555	65,369	0.077	94.5
Apparent CP digestibility (n = 25 per group), %				
D20–32	66.8	74.3	0.001	1.22
D33–42	77.6	80.6	0.055	0.99

^1^ C—control group fed complete mixtures without halloysite, E—experimental group fed complete mixtures with 1% of halloysite added.

**Table 3:** Influence of chickens’ dietary halloysite supplementation on quality of air and litter on days of rearing 21 and 35 (D21 and D35, respectively).

Treatment ^1^	C		E	
Day of Rearing	D21	D35	D21	D35
Weather condition (n = 20)				
Air temperature outside, °C	+5.8 ± 0.56	−9.9 ± 1.03	+6 ± 0.64	−9.8 ± 0.87
RH outside, %	78 ± 0.7	82 ± 3.1	78 ± 0.9	82 ± 3.2
Air parameters inside building (n = 15 per group)				
Air bulb temperature, °C	24.4 ± 0.23 ^b^	18.7 ± 0.57 ^a^	25.1 ± 0.33 ^b^	19.3 ± 0.57 ^a^
Air velocity, ms^−1^	0.4 ± 0.04	0.2 ± 0.03	0.3 ± 0.05	0.3 ± 0.03
Cooling rate, mWcm^−2^	34.7 ± 1.72 ^a,b^	38.8 ± 2.57 ^a,b^	31.0 ± 2.17 ^a^	41.9 ± 2.41 ^b^
Air relative humidity, %	57 ± 2.5 ^b^	82 ± 3.5 ^c^	49 ± 2.6 ^a^	83 ± 3.5 ^c^
Concentration of CO_2_, ppm	3090 ± 149.9 ^a^	5742 ± 207.5 ^b^	3079 ± 207.5 ^a^	5250 ± 227.3 ^b^
Concentration of NH_3_, ppm	4.4 ± 0.93 ^a^	23.2 ± 1.26 ^c^	7.2 ± 1.26 ^a,b^	10.9 ± 1.38 ^b^
Litter parameters (n = 15 per group)				
Temperature on litter surface, °C	26.1 ± 0.19 ^b^	26.6 ± 0.23 ^b^	25.1 ± 0.38 ^a^	24.7 ± 0.34 ^a^
Litter temperature at a depth of 3 cm, °C	27 ± 0.50 ^a^	28.8 ± 0.70 ^b^	28.0 ± 1.17 ^a^	29.1 ± 1.36 ^b^
Litter dry matter, %	58.8 ± 2.43 ^a^	63.3 ± 2.87 ^a,b^	59.6 ± 2.37 ^a,b^	64.6 ± 1.31 ^b^
Litter pH	6.7 ± 0.27	6.8 ± 0.38	7.4 ± 0.10	6.7 ± 0.25
Concentration of NH_3_ in litter, ppm	19.1 ± 1.79 ^a,b^	16.3 ± 2.16 ^a^	18 ± 1.22 ^a^	22.4 ± 3.82 ^b^

^1^ C—control group fed complete mixtures without halloysite, E—experimental group fed complete mixtures with 1% of halloysite added; ^a,b,c^ Values in the rows marked with various letters differ significantly (*p* < 0.05).

## Data Availability

The data presented in this study are available on request from the corresponding author.
